# Supplementary material for: The role of transposable elements in the evolution of non-mammalian vertebrates and invertebrates
Source: Genome Biol. 2010 Jun 2;11(6):R59. doi: 10.1186/gb-2010-11-6-r59 (PMC2911107; doi:10.1186/gb-2010-11-6-r59)
Supplement: Additional file 1 — Number of sense/antisense TE insertions within intronic sequences. [file gb-2010-11-6-r59-S1.DOC]

**Table S1:** number of sense/antisense TE insertions within intronic sequences

| species | TE | total | Sense | antisense | χ^2^ p-value |
| --- | --- | --- | --- | --- | --- |
| *G. Gallus* | SINE | - | - | - | - |
|  | LINE | 65, 035 | 32, 763 | 32,272 | 0.17 |
|  | LTR | 7553 | 3401 | 4152 | 9.90e-10 |
|  | DNA | 6554 | 2915 | 3639 | 2.65e-10 |
| *D. rerio* | SINE | 113926 | 58186 | 55740 | 3.04e-7 |
|  | LINE | 37228 | 16612 | 20616 | 7.32e-49 |
|  | LTR | 21496 | 10128 | 11368 | 2.32e-9 |
|  | DNA | 585408 | 291502 | 293906 | 0.026 |
| *C. intestinalis* | SINE | 20360 | 10607 | 9753 | 0.000024 |
|  | LINE | 11172 | 5632 | 5540 | 0.54 |
|  | LTR | 112 | 56 | 56 | 1 |
|  | DNA | 22056 | 10934 | 11122 | 0.375 |
| *D. melanogaster* | SINE | - | - | - | - |
|  | LINE | 2964 | 1295 | 1669 | 0.0000012 |
|  | LTR | 5394 | 2560 | 2834 | 0.0087 |
|  | DNA | 5560 | 2613 | 2947 | 0.0016 |
| *C. elegans* | SINE | 243 | 110 | 133 | 0.317 |
|  | LINE | 103 | 37 | 66 | 0.0486 |
|  | LTR | 137 | 75 | 62 | 0.468 |
|  | DNA | 17724 | 9025 | 8699 | 0.0852 |
