# Supplementary material for: The role of transposable elements in the evolution of non-mammalian vertebrates and invertebrates
Source: Genome Biol. 2010 Jun 2;11(6):R59. doi: 10.1186/gb-2010-11-6-r59 (PMC2911107; doi:10.1186/gb-2010-11-6-r59)
Supplement: Additional file 2 — Number of exonizations versus number of ESTs. [file gb-2010-11-6-r59-S2.DOC]

**Table S2**: number of exonizations versus number of ESTs

| Species | Number of ESTs | Number of exonizations |
| --- | --- | --- |
| *Gallus gallus* | 599,785 | 70 |
| *Danio rerio* | 1,380,071 | 153 |
| *Ciona intestinalis* | 1,205,674 | 12 |
| *Drosophila melanigaster* | 573,981 | 0 |
| *C. elegans* | 352,044 | 4 |
