# Supplementary material for: The role of transposable elements in the evolution of non-mammalian vertebrates and invertebrates
Source: Genome Biol. 2010 Jun 2;11(6):R59. doi: 10.1186/gb-2010-11-6-r59 (PMC2911107; doi:10.1186/gb-2010-11-6-r59)
Supplement: Additional file 3 — Number of exonizations found in the coding sequence or UTR (within annotated genes). [file gb-2010-11-6-r59-S3.DOC]

**Table S3:** Number of exonization found in the CDS or UTR (in these found within annotated genes)

| Species | 5' UTR | 3' UTR | CDS | CDS divisible by 3 |
| --- | --- | --- | --- | --- |
| *G. gallus* | 7 | 0 | 14 (66%) | 7 (50%) |
| *D. rerio* | 10 | 1 | 36 (76%) | 14 (38%) |
| *C. intestinalis* | 1 | 0 | 7 (87%) | 3 (42.8%) |
