# Supplementary material for: The role of transposable elements in the evolution of non-mammalian vertebrates and invertebrates
Source: Genome Biol. 2010 Jun 2;11(6):R59. doi: 10.1186/gb-2010-11-6-r59 (PMC2911107; doi:10.1186/gb-2010-11-6-r59)
Supplement: Additional file 4 — Sequences of the internal D. melanogaster exons with TE insertions. [file gb-2010-11-6-r59-S4.DOC]

**Table S5:** average length and average TE percentage in the last exon of 7 species.

|  | *H. sapiens* | *M. musculus* | *G. gallus* | *D. rerio* | *C. intestinalis* | *D. melanogaster* | *C. elegans* |
| --- | --- | --- | --- | --- | --- | --- | --- |
| length | 1300 | 1189 | 915.04 | 701.29 | 663.6 | 827.4 | 286.89 |
| TE percentage | 7.6 | 1.7 | 0.39 | 3.37% | 1.32 | 0.37 | 0.79 |
