# Supplementary material for: The role of transposable elements in the evolution of non-mammalian vertebrates and invertebrates
Source: Genome Biol. 2010 Jun 2;11(6):R59. doi: 10.1186/gb-2010-11-6-r59 (PMC2911107; doi:10.1186/gb-2010-11-6-r59)
Supplement: Additional file 5 — Average lengths and average TE percentages in first exons. [file gb-2010-11-6-r59-S5.DOC]

**Text S1**: The sequences of the internal *Drosophila melanogaster* exons with TEs insertions.

>nej

CTCAACTCAACAGTC

TGCCCTACGGAGTGGGTCAGTATGGTGGCCCAGGCGGTGGTAACAATCCT

CAGCAACAGCAGCAGCAACAGCAGCAACAACTTCTCGCCCAGCAGATGGC

CCAAAGAGGTGGCGTCGTACCGGGCATGCCGCAGGGTAATCGGCCCGTTG

GCACAGTGGTGCCCATGTCCACACTCGGCGGCGATGGATCAGGGCCCGCG

GGGCAGCTGGTAAGCGGGAATCCTCAGCAGCAGCAGATGCTGGCGCAGCA

GCAAACCGGAGCCATGGGCCCGCGTCCTCCGCAACCAAACCAGCTGCTCG

GTCATCCCGGCCAGCAGCAGCAGCAGCAACAGCAGCCTGGCACCTCGCAG

CAGCAGCAACAGCAGCAGGGAGTCGGAATCGGAGGAGCAGGCGTTGTGGC

CAATGCAGGAACCGTGGCTGGCGTGCCGGCAGTGGCAGGCGGCGGAGCCG

GTGGTGCCGTACAATCTAGCGGCCCTGGTGGCGCCAATCGCGATGTGCCC

GACGACCGTAAGCGACAGATCCAGCAGCAACTGATGCTGCTCCTCCATGC

ACACAAATGCAATCGCAGGGAGAACCTGAATCCGAACAGGGAAGTGTGCA

ACGTTAACTACTGCAAGGCGATGAAATCCGTGCTGGCCCACATGGGCACT

TGCAAACAGAGCAAGGACTGCACCATGCAGCATTGTGCCTCTTCGCGCCA

AATTCTGTTGCATTATAAAACGTGCCAGAACAGTGGCTGCGTCATTTGCT

ATCCCTTCCGGCAGAATCATTCGGTTTTTCAAAATGCGAATGTGCCGCCA

GGAGGCGGACCGGCAGGAATTGGAGGTGCGCCACCAGGTGGCGGCGGAGC

GGGTGGTGGAGCGGCTGGAGCAGGCGGTAATCTTCAGCAGCAACAGCAGC

AGCAACAACAGCAGCAGCAGAACCAGCAGCCCAATCTGACGGGTCTGGTA

GTGGATGGCAAGCAAGGACAGCAGGTTGCACCGGGAGGTGGCCAAAATAC

TGCCATAGTTCTTCCCCAGCAACAGGGAGCGGGCGGTGCACCGGGTGCGC

CGAAAACGCCTGCGGATATGGTGCAACAATTGACCCAACAGCAGCAGCAG

CAGCAACAGCAGGTTCACCAGCAACAGGTTCAGCAACAGGAACTCCGTCG

ATTCGATGGCATGAGCCAGCAAGTCGTAGCAGGTGGTATGCAACAGCAGC

AGCAGCAGGGTTTGCCTCCTGTGATTCGCATTCAAGGCGCTCAGCCGGCC

GTCAGGGTACTGGGACCAGGTGGTCCCGGCGGCCCAAGTGGACCAAATGT

TCTGCCGAACGATGTTAACAGCCTGCATCAACAACAGCAACAAATGCTGC

AACAGCAGCAGCAACAGGGCCAGAATCGACGACGCGGTGGCCTGGCCACC

ATGGTGGAGCAACAACAGCAGCATCAGCAACAACAGCAGCAACCCAATCC

CGCCCAGCTGGGTGGCAACATTCCAGCACCACTCTCTGTCAACGTCGGTG

GCTTTGGCAATACCAATTTCGGTGGTGCAGCTGCCGGCGGAGCCGTGGGA

GCCAACGATAAGCAGCAACTGAAGGTGGCCCAAGTGCATCCGCAGAGCCA

TGGCGTAGGAGCGGGCGGTGCATCAGCGGGCGCCGGGGCGAGTGGTGGTC

AAGTGGCAGCCGGTTCCAGTGTCCTGATGCCAGCCGATACCACGGGCAGT

GGTAATGCGGGCAATCCCAACCAGAATGCAGGCGGTGTAGCTGGAGGTGC

CGGCGGTGGCAATGGCGGAAACACTGGACCTCCGGGCGACAACGAGAAAG

ACTGGCGGGAATCGGTGACCGCCGATCTGCGCAACCACCTCGTCCACAAA

CTGGTGCAGGCCATCTTCCCCACCTCGGATCCTACGACCATGCAGGACAA

ACGGATGCATAATCTCGTTTCATACGCGGAAAAGGTCGAGAAGGACATGT

ACGAAATGGCCAAGTCCAGATCGGAGTACTATCACCTGCTGGCCGAGAAG

ATCTACAAGATTCAAAAGGAGCTGGAGGAGAAGCGACTGAAGCGTAAGGA

GCAGCATCAGCAGATGCTGATGCAGCAACAGGGCGTTGCGAATCCAGTGG

CTGGAGGAGCGGCTGGCGGAGCAGGCAGTGCAGCTGGTGTAGCGGGCGGT

GTAGTCTTGCCCCAGCAGCAACAGCAGCAGCAACAACAACAGCAGCAGCA

GGGTCAGCAGCCTCTGCAGAGCTGTATCCATCCAAGCATCAGTCCAATGG

GCGGTGTGATGCCGCCGCAGCAGCTGCGTCCACAGGGACCACCTGGAATA

CTGGGCCAACAGACGGCAGCAGGCCTGGGCGTCGGCGTGGGAGTGACCAA

CAATATGGTTACCATGCGCAGTCATTCGCCCGGTGGCAACATGCTCGCCT

TGCAGCAACAACAGCGCATGCAGTTCCCGCAACAACAGCAGCAACAACCG

CCAGGGTCTGGAGCCGGCAAAATGCTGGTCGGTCCACCAGGACCCAGTCC

CGGTGGCATGGTGGTCAATCCCGCGCTCTCGCCTTACCAGACGACCAATG

TGCTCACCAGTCCGGTGCCAGGACAGCAGCAACAGCAGCAGTTCATTAAT

GCGAACGGCGGCACTGGCGCCAATCCTCAACTGAGCGAAATCATGAAGCA

GCGTCACATTCACCAGCAGCAGCAGCAACAACAACAGCAGCAGCAGCAGG

GAATGTTGTTGCCGCAGTCGCCATTTAGCAATTCAACACCTCTACAACAA

CAACAGCAGCAGCAGCAGCAGCAACAACAGCAGCAGCAGGCGACTAGCAA

CAGTTTTAGCTCACCAATGCAGCAACAGCAGCAAGGTCAGCAACAGCAAC

AACAGAAGCCCGGCAGTGTGCTGAATAATATGCCGCCCACGCCCACGAGT

CTGGAAGCCCTGAATGCGGGGGCCGGAGCGCCGGGAACTGGAGGATCCGC

CTCCAATGTAACGGTTTCAGCTCCGAGCCCATCGCCTGGCTTCTTGTCCA

ACGGCCCGTCGATTGGCACGCCCTCCAACAATAATAATAATAGTAGTGCT

AACAACAACCCGCCCTCGGTGAGCAGTCTAATGCAACAGCCGCTGAGCAA

TCGGCCGGGTACGCCTCCTTACATACCCGCTTCCCCAGTGCCGGCGACAA

GTGCCTCCGGATTAGCGGCGAGCAGTACGCCCGCATCAGCAGCAGCCACC

TGTGCGAGTAGTGGCAGTGGCAGCAATAGCAGCAGCGGAGCAACTGCAGC

GGGTGCAAGTTCCACGTCATCATCTTCCTCGGCGGGCTCGGGTACACCAC

TCAGCTCGGTATCGACTCCTACATCGGCCACGATGGCCACCAGCAGCGGT

GGTGGTGGTGGTGGTGGGGGCAATGCAGGAGGCGGATCATCCACTACGCC

CGCTAGCAATCCACTGCTCCTCATGTCTGGAGGAACGGCAGGAGGCGGAA

CGGGAGCAACGACCACCACATCGACATCCTCGAGCAGTCGCATGATGAGC

AGCTCCAGCAGTCTCTCCTCACAGATGGCTGCCCTGGAGGCTGCGGCGCG

AGACAACGACGATGAGACGCCCTCGCCATCCGGCGAGAATACGAACGGCA

GTGGTGGCAGTGGAAATGCCGGCGGTATGGCCTCCAAGGGCAAACTGGAC

TCCATTAAGCAAGATGATGATATCAAGAAGGAGTTTATGGATGACAGCTG

TGGCGGAAATAACGATAGCTCGCAGATGGATTGCTCGACGGGTGGTGGCA

AGGGCAAGAATGTGAACAACGACGGAACAAGCATGATCAAAATGGAGATC

AAGACGGAGGATGGACTCGATGGCGAGGTAAAGATCAAAACGGAGGCCAT

GGATGTGGACGAGGCTGGAGGATCGACAGCCGGAGAGCATCATGGCGAAG

GTGGCGGCGGCAGTGGTGTTGGCGGCGGTAAGGATAACATAAATGGTGCG

CACGATGGCGGAGCGACAGGCGGTGCTGTGGACATAAAACCCAAGACGGA

GACGAAACCACTCGTACCGGAGCCACTGGCACCCAATGCAGGTGACAAGA

AAAAGAAGTGCC

>nej_exon_repeatmasker

CTCAACTCAACAGTCTGCCCTACGGAGTGGGTCAGTATGGTGGCCCAGGC

GGTGGTAACAATCCTCAGCAACAGCAGCAGCAACAGCAGCAACAACTTCT

CGCCCAGCAGATGGCCCAAAGAGGTGGCGTCGTACCGGGCATGCCGCAGG

GTAATCGGCCCGTTGGCACAGTGGTGCCCATGTCCACACTCGGCGGCGAT

GGATCAGGGCCCGCGGGGCAGCTGGTAAGCGGGAATCCTCAGCAGCAGCA

GATGCTGGCGCAGCAGCAAACCGGAGCCATGGGCCCGCGTCCTCCGCAAC

Simple repeat

CAAACCAGCTGCTCGGTCATCCCGGCcagcagcagcagcagcaacagcag

cctggcacctcgcagcagcagcaacagcagcagGGAGTCGGAATCGGAGG

AGCAGGCGTTGTGGCCAATGCAGGAACCGTGGCTGGCGTGCCGGCAGTGG

CAGGCGGCGGAGCCGGTGGTGCCGTACAATCTAGCGGCCCTGGTGGCGCC

AATCGCGATGTGCCCGACGACCGTAAGCGACAGATCCAGCAGCAACTGAT

GCTGCTCCTCCATGCACACAAATGCAATCGCAGGGAGAACCTGAATCCGA

ACAGGGAAGTGTGCAACGTTAACTACTGCAAGGCGATGAAATCCGTGCTG

GCCCACATGGGCACTTGCAAACAGAGCAAGGACTGCACCATGCAGCATTG

TGCCTCTTCGCGCCAAATTCTGTTGCATTATAAAACGTGCCAGAACAGTG

GCTGCGTCATTTGCTATCCCTTCCGGCAGAATCATTCGGTTTTTCAAAAT

GCGAATGTGCCGCCAGGAGGCGGACCGGCAGGAATTGGAGGTGCGCCACC

AGGTGGCGGCGGAGCGGGTGGTGGAGCGGCTGGAGCAGGCGGTAATCTTc

Simple repeat

agcagcaacagcagcagcaacaacagcagcagcagaaccagcagcCCAAT

CTGACGGGTCTGGTAGTGGATGGCAAGCAAGGACAGCAGGTTGCACCGGG

AGGTGGCCAAAATACTGCCATAGTTCTTCCCCAGCAACAGGGAGCGGGCG

GTGCACCGGGTGCGCCGAAAACGCCTGCGGATATGGTGCAACAATTGACC

caacagcagcagcagcagcaacagcaggttcaccagcaacaggttcagca

acaggaactccgtcgattcgatggcatgagccagcaagtcgtagcaggtg

gtatgcaacagcagcagcagcagGGTTTGCCTCCTGTGATTCGCATTCAA

GGCGCTCAGCCGGCCGTCAGGGTACTGGGACCAGGTGGTCCCGGCGGCCC

AAGTGGACCAAATGTTCTGCCGaacgatgttaacagcctgcatcaacaac

agcaacaaatgctgcaacagcagcagcaacagggccagaatcgacgacgc

ggtggcctggccaccatggtggagcaacaacagcagcatcagcaacaaca

gcagcaacCCAATCCCGCCCAGCTGGGTGGCAACATTCCAGCACCACTCT

CTGTCAACGTCGGTGGCTTTGGCAATACCAATTTCGGTGGTGCAGCTGCC

GGCGGAGCCGTGGGAGCCAACGATAAGCAGCAACTGAAGGTGGCCCAAGT

GCATCCGCAGAGCCATGGCGTAGGAGCGGGCGGTGCATCAGCGGGCGCCG

GGGCGAGTGGTGGTCAAGTGGCAGCCGGTTCCAGTGTCCTGATGCCAGCC

GATACCACGGGCAGTGGTAATGCGGGCAATCCCAACCAGAATGCAGGCGG

TGTAGCTGGAGGTGCCGGCGGTGGCAATGGCGGAAACACTGGACCTCCGG

GCGACAACGAGAAAGACTGGCGGGAATCGGTGACCGCCGATCTGCGCAAC

CACCTCGTCCACAAACTGGTGCAGGCCATCTTCCCCACCTCGGATCCTAC

GACCATGCAGGACAAACGGATGCATAATCTCGTTTCATACGCGGAAAAGG

TCGAGAAGGACATGTACGAAATGGCCAAGTCCAGATCGGAGTACTATCAC

CTGCTGGCCGAGAAGATCTACAAGATTCAAAAGGagctggaggagaagcg

actgaagcgtaaggagcagcatcagcagatgctgatgcagcaacagggcg

ttgcgaatccagtggctggaggagcggctggcggagcaggcagtgcagct

ggtgtagcgggcggtgtagtcttgccccagcagcaacagcagcagcaaca

acaacagcagcagcagggtcagcagcctctgcagagctgtatccatccaa

TART#LINE/tel

gcatcagtccaatgggcggtgtgatgccgccgcagcagctgcgtccacag

ggaccacctggaatactgggccaacagacggcagcaggcctgggcgtcgg

cgtgggagtgaccaacaatatggttaccatgcgcagtcattcgcccggtg

gcaacatgctcgccttgcagcaacaacagcgcatgcagttcccgcaacaa

cagcagcaacaACCGCCAGGGTCTGGAGCCGGCAAAATGCTGGTCGGTCC

ACCAGGACCCAGTCCCGGTGGCATGGTGGTCAATCCCGCGCTCTCGCCTt

accagacgaccaatgtgctcaccagtccggtgccaggacagcagcaacag

cagcagttcattaatgcgaacggcggcactggcgccaatcctcaactgag

cgaaatcatgaagcagcgtcacattcaccagcagcagcagcaacaacaac

agcagcagcagcagggaatgttgttgccgcagtcgccatttagcaattca

acacctctacaacaacaacagcagcagcagcagcagcaacaacagcagca

TART_B1#LINE

gcaggcgactagcaacagttttagctcaccaatgcagcaacagcagcaag

gtcagcaacagcaacaacagaagcccggcagtgtgctgaataatatgccg

cccacgcccacgagtctggaagccctgaatgcgggggccggagcgccggg

aactggaggatccgcctccaatgtaacggtttcagctccgagcccatcgc

ctggcttcttgtccaacggcccgtcgattggcacgccctccaacaataat

aataatagtagtgctaacaacAACCCGCCCTCGGTGAGCAGTCTAATGCA

ACAGCCGCTGAGCAATCGGCCGGGTACGCCTCCTTACATACCCGCTTCCC

CAGTGCCGGCGACAAGTGCCTCCGGATTAGCGGCGAGCAGTACGCCCgca

Simple repeat

tcagcagcagccacctgtgcgagtagtggcagtggcagcaatagcagcag

cggagcaactgcagcggGTGCAAGTTCCACGTCATCATCTTCCTCGGCGG

GCTCGGGTACACCACTCAGCTCGGTATCGACTCCTACATCGGCCACGATG

GCCACCAGCAGCggtggtggtggtggtggtggGGGCAATGCAGGAGGCGG

ATCATCCACTACGCCCGCTAGCAATCCACTGCTCCTCATGTCTGGAGGAA

CGGCAGGAGGCGGAACGGGAGCAACGACCACCACATCGACATCCTCGAGC

AGTCGCATGATGAGCAGCTCCAGCAGTCTCTCCTCACAGATGGCTGCCCT

GGAGGCTGCGGCGCGAGACAACGACGATGAGACGCCCTCGCCATCCGGCG

AGAATACGAACGGCAGTGGTGGCAGTGGAAATGCCGGCGGTATGGCCTCC

AAGGGCAAACTGGACTCCATTAAGCAAGATGATGATATCAAGAAGGAGTT

TATGGATGACAGCTGTGGCGGAAATAACGATAGCTCGCAGATGGATTGCT

CGACGGGTGGTGGCAAGGGCAAGAATGTGAACAACGACGGAACAAGCATG

ATCAAAATGGAGATCAAGACGGAGGATGGACTCGATGGCGAGGTAAAGAT

CAAAACGGAGGCCATGGATGTGGACGAGGCTGGAGGATCGACAGCCGGAG

AGCATCATGGCGAAGGTGGCGGCGGCAGTGGTGTTGGCGGCGGTAAGGAT

AACATAAATGGTGCGCACGATGGCGGAGCGACAGGCGGTGCTGTGGACAT

AAAACCCAAGACGGAGACGAAACCACTCGTACCGGAGCCACTGGCACCCA

ATGCAGGTGACAAGAAAAAGAAGTGCC

>cno

ACCGCCGATTGCCGC

ACATGCCGCCATGAACGCTTACAATGGCAGTTCCCCGCTTGCACCGCAGC

AGCAACCACAGCAGCAACAGCAATCGCCATACCAGCAGCAGCAGCAGCAG

CAACATATGCAGGCCAATGCTAACCTCCCGCCCACGCGACCCGTCTCTGC

CTATTACCACAGCCAGCAGTCGGCGCAGCAGCAGCTGCAGCAACAACAGC

AGCAGCAGCAACAACATTCCCTGCAGCAGCAACAGTTCGCGCTCAGCAGT

GGCAATCTCAATGGCCAGCAACAGCAACAGCAACAGCACCAGCACCAGCA

CCAGCTCACCTTGAACAACCGCACCAAGAGCCAGCAGAACTTCCAGCACA

CTCTTCGCATGCAGCAGATGATGGCTCCCTCCATGCCTAACATCAGCAAC

ATGTACCATCACCAGCAGCAACAACAGCAACAGCTGCCCCTGCAACAGCA

GCAGCAGCAGCAACAGCAACCTCTGATGAGCAGCAGTCAGAGCATGCAGA

ATGTGAACGATTTCGCAGGTGGCTATCAGAATGGCAGTTTGGAGTACAGG

CGCAGTCAGCTCCATGATCCATCCACGCTCTACGAAATTCAGCAGCAACA

GTTGCAACAGCAACAGCAACAGCAACAGCAACAGCAACAGCAGCAGCAGG

CATCGCCGAATTTTATAGCCCTGCCCCCTAAACCATTGGGCAGCTTACAG

TCTCCCAATAAACCAAATGTTCCACCAAGTACAGCACCAAAACCGCAGCA

GGACAAGCCGCCTCTACCGCCTACAGCAACGCATCCGCTGTTCAAAGCCA

CGCAGCAGATTGCTCCGGGAATGAACTACGTAGCCAGTACATTGGATCCG

CCAAAGGGCAGCTATGTGGCGTCCAATCAAGGGAACAATCGTCCCCTTCA

CAGCGGTAGCAATCCATGGGAAAGGGAGGAGCGTGAGAAGGATTTAGAGA

TGCGTCGCGAACACATTCGCCAGTGGCGGGAGCAGCAGATATCAGAGCTG

TCCCAGATCGTATCCCGTTCCCCAATGCAAGAGGAGCAACTGAAAACCCT

GATACTCGAGCGCGACTTTGAGCGAAGAGCACAAGAACTACAAGAGCAAG

AGGAACAGGATCAGGAACAGCAGTACGACAAGGAGAACGTCCAGGAGTTG

TTCAGATTGGCTGGCGGCGGTCAGGTCAGTGCCATACAAACACCCATTAC

CAGTTACCGACAAACGGAGATAAAACTGGCGGAGATGCCAGACAGCAACA

GTCTGGTGGATTCAGTGCCACCACAGCCACCAGCTCCAACTGCCCAGCCG

CTGAGCAGCAACACACAGCAGCCCAAGAGTATACTTAAGCACAATCGGTA

TTCCGAAGGCGGGGTTGGTCCCAGTGGGGCACCCTCATCGCCATCGAAGT

CGCAGAAATCCGCAAGTTTTGCGGACGAGCGACATCTGCACACTGAGCAT

CCGATATCTAATCTAGCGAAGGAGCTTAATCAGCTCACAATGCTTGATAA

GGACAATAACAACGAGACTCTGGATGCTGTTGTGCCGCCACCTCCACCGC

CAGAAAGGAACAGTTCTTATTTGATCATGTCACAGCAGAAACTGCGAGGC

AGCACTGGAAACGCGACCTCGTCCATGGGCCTACTGAAAACGGCCACCAG

CAATCAGGCCGCAGCTGCCGTAGAGATTAAAAAGGCATCTCTTCTCAACA

CTCAAACTAATAATAACAACAATTTGAGTGGGAGCTTAAACAACAACACC

ATGCATGGATCACCACTCAGCGCCATGGAGCTAAATGCCGCCTATGTTTC

GGCCACGGGAACAGGAATAATTGGAGGATTGGGCACACCGCCTCCGCCGC

CACCGTTGATGCAAAGGGACAACAAGCGGGTGAGCTTCCATGATGAAGAG

AATAATTTTGTGAGCGGGAATAGCCAGCAACAACAGCTGCAGCAGCAATA

TATTATGGATAATTACGGTATGGAGCATCAGGATTTGGACACTATCAGAG

AGGATACGAGT

>cno

ACCGCCGATTGCCGCACATGCCGCCATGAACGCTTACAATGGCAGTTCCC

CGCTTGCACCgcagcagcaaccacagcagcaacagcaatcgccataccag

cagcagcagcagcagcaacatatgcagGCCAATGCTAACCTCCCGCCCAC

GCGACCCGTCTCTGCCTATTACCACAGCCAGCAGTCGGCgcagcagcagc

tgcagcaacaacagcagcagcagcaacaacaTTCCCTGcagcagcaacag

ttcgcgctcagcagtggcaatctcaatggccagcaacagcaacagcaaca

gcaccagcaccagcaccagctcaccttgaacaaccgcaccaagagccagc

agaacttccagcacactcttcgcatgcagcagatgatggctccctccatg

TART_DV#LINE

cctaacatcagcaacatgtaccatcaccagcagcaacaacagcaacagct

gcccctgcaacagcagcagcagcagcaacagcaacctctgatgagcagca

gtcagagcatgcagaatgtgaacgatttcgcaggtggctatcagaatggc

agtttggagtacaggcgcagtcagctccatgatccatccacgctctacga

aattcagcagcaacagttgcaacagcaacagcaacagcaacagcaacagc

aacagcagcagcagGCATCGCCGAATTTTATAGCCCTGCCCCCTAAACCA

TTGGGCAGCTTACAGTCTCCCAATAAACCAAATGTTCCACCAAGTACAGC

ACCAAAACCGCAGCAGGACAAGCCGCCTCTACCGCCTACAGCAACGCATC

CGCTGTTCAAAGCCACGCAGCAGATTGCTCCGGGAATGAACTACGTAGCC

AGTACATTGGATCCGCCAAAGGGCAGCTATGTGGCGTCCAATCAAGGGAA

CAATCGTCCCCTTCACAGCGGTAGCAATCCATGGGAAAGGGAGGAGCGTG

AGAAGGATTTAGAGATGCGTCGCGAACACATTCGCCAGTGGCGGGAGCAG

CAGATATCAGAGCTGTCCCAGATCGTATCCCGTTCCCCAATGCAAGAGGA

GCAACTGAAAACCCTGATACTCGAGCGCGACTTTGAGCGAAGAGCACAAG

AACTACAAGAGCAAGAGGAACAGGATCAGGAACAGCAGTACGACAAGGAG

AACGTCCAGGAGTTGTTCAGATTGGCTGGCGGCGGTCAGGTCAGTGCCAT

ACAAACACCCATTACCAGTTACCGACAAACGGAGATAAAACTGGCGGAGA

TGCCAGACAGCAACAGTCTGGTGGATTCAGTGCCACCACAGCCACCAGCT

CCAACTGCCCAGCCGCTGAGCAGCAACACACAGCAGCCCAAGAGTATACT

TAAGCACAATCGGTATTCCGAAGGCGGGGTTGGTCCCAGTGGGGCACCCT

CATCGCCATCGAAGTCGCAGAAATCCGCAAGTTTTGCGGACGAGCGACAT

CTGCACACTGAGCATCCGATATCTAATCTAGCGAAGGAGCTTAATCAGCT

CACAATGCTTGATAAGGACAATAACAACGAGACTCTGGATGCTGTTGTGC

CGCCACCTCCACCGCCAGAAAGGAACAGTTCTTATTTGATCATGTCACAG

CAGAAACTGCGAGGCAGCACTGGAAACGCGACCTCGTCCATGGGCCTACT

GAAAACGGCCACCAGCAATCAGGCCGCAGCTGCCGTAGAGATTAAAAAGG

CATCTCTTCTCAACACTCAAACTAATAATAACAACAATTTGAGTGGGAGC

TTAAACAACAACACCATGCATGGATCACCACTCAGCGCCATGGAGCTAAA

TGCCGCCTATGTTTCGGCCACGGGAACAGGAATAATTGGAGGATTGGGCA

CACCGCCTCCGCCGCCACCGTTGATGCAAAGGGACAACAAGCGGGTGAGC

TTCCATGATGAAGAGAATAATTTTGTGAGCGGGAATAGCCAGCAACAACA

GCTGCAGCAGCAATATATTATGGATAATTACGGTATGGAGCATCAGGATT

TGGACACTATCAGAGAGGATACGAGT

>CG14821

GGTCCCGA

CTACGATTCG GCCAGCGAGA CGGAGATCGC ATTGGAGGAG GCCCGACTAA

ACGCCCTGCG TCAACATTGG GAGTCCGCCA GCATTACGGC CTCCATCTGC

TCCTCGGCCA GCCGCTCGCT GCAGACGACG CCCATCCGCC AGGTGCAGGT

GCCATTGAAG CAGCCACCGC GTGGCGCCGG CCTGCTGATT CAGCCTTCTA

GCGCCTCGGC GGTCACCGCC TCCACCTCCT CCTCCTCCTC ATCGACGATA

GTCGCGCTGC CCACGACGGA GTATCTACCT CAGCATGTGT TGACGGGACG

GCAAGTGGTG CAGCAGCAGC AACAACAGCA GCAGCAACAG GCGGCAGTGC

TCTACCAGCA GCAGCAGCAG CAACTAATAC TGCTGCCCCA GTTGGATCCT

AATGTTGTCA ACACGTCCAC GGCAGCAGCA GCGGCACAGC TCGTCCAGTT

GCAGCAGCAG CATCAACAGC AGCAACAACA GCTACAGCTG CAGCAGCAAC

AGCAGCTGCA ACTCAATCAG CAGCAGCAGC AGCAACTTTA CCAGCAGCAG

CAACAGCAAT ACCTGCTGAC CCTTCCCGCC GGATCAAAGC CCCAGAGCGT

GAGTCCAAAG CGCCTGATCT ATGGTGGACC CGCTGCAACC ACTCTCCTCT

GTGCAACTGG

>CG14821

GGTCCCGACTACGATTCGGCCAGCGAGACGGAGATCGCATTGGAGGAGGC

CCGACTAAACGCCCTGCGTCAACATTGGGAGTCCGCCAGCATTACGGCCT

CCATCTGCTCCTCGGCCAGCCGCTCGCTGCAGACGACGCCCATCCGCCAG

GTGCAGGTGCCATTGAAGCAGCCACCGCGTGGCGCCGGCCTGCTGATTCA

GCCTTCTAGCGCCTCGGCGGTCACCGCCTCCACCTCCTCCTCCTCCTCAT

CGACGATAGTCGCGCTGCCCACGACGGAGTATCTACCTCAGCATGTGTTG

ACGGGACGGCAAGTGGTGCAGcagcagcaacaacagcagcagcaacaggc

ggcagtgctctaccagcagcagcagcagcaactaatactgctgccccagt

tggatcctaatgttgtcaacacgtccacggcagcagcagcggcacagctc

TART_DV#LINE

gtccagttgcagcagcagcatcaacagcagcaacaacagctacagctgca

gcagcaacagcagctgcaactcaatcagcagcagcagcagcaactttacc

agcagcagcaacagcaataccTGCTGACCCTTCCCGCCGGATCAAAGCCC

CAGAGCGTGAGTCCAAAGCGCCTGATCTATGGTGGACCCGCTGCAACCAC

TCTCCTCTGTGCAACTGG
