# Supplementary material for: The role of transposable elements in the evolution of non-mammalian vertebrates and invertebrates
Source: Genome Biol. 2010 Jun 2;11(6):R59. doi: 10.1186/gb-2010-11-6-r59 (PMC2911107; doi:10.1186/gb-2010-11-6-r59)
Supplement: Additional file 6 — Average lengths and average TE percentages in last exons. [file gb-2010-11-6-r59-S6.DOC]

**Table S4:** average length and average TE percentage in the first exon of 7 species.

|  | *H. sapiens* | *M. musculus* | *G. gallus* | *D. rerio* | *C. intestinalis* | *D. melanogaster* | *C. elegans* |
| --- | --- | --- | --- | --- | --- | --- | --- |
| length | 464.6 | 392.7 | 202.19 | 249.06 | 283.6 | 339.6 | 162.9 |
| TE percentage | 3.5 | 0.4 | 0.11 | 0.34 | 0.24 | 0.29 | 0.6 |
